# Clinical Study of Biostimulation with Low-Power Diode Laser After Dental Extractions

**DOI:** 10.3390/clinpract15050090

**Published:** 2025-05-06

**Authors:** Yolanda Collado Murcia, Pia Lopez-Jornet, Francisco Parra Perez

**Affiliations:** Facultad de Medicina y Odontologia, Departament Oral Medicine Hospital Morales Meseguer, Clínica Odontológica Universitaria, Adv Marques de los Velez s/n, 30098 Murcia, Spain; yolanda.collado1@um.es (Y.C.M.); francisco.parra1@um.es (F.P.P.)

**Keywords:** pain, photobiomodulation, swelling, tooth extraction

## Abstract

**Introduction:** The objective of the present work is to assess the efficacy of photobiomodulation (PBM) with respect to pain, inflammation, and healing after tooth extractions as compared with a sham treatment. **Method:** A single-blinded, randomized clinical study conducted in a private dental clinic in Murcia, it included 124 patients who needed a tooth extraction, excluding those with medical conditions that could affect healing (such as non-controlled diabetes, immunosuppression, or hemorrhagic disorders). Group I (Experimental): extraction and PBM session with a diode laser (power: 0.5 W, energy 15 J/cm^2^ for 10–30 s at 1 mm from the tissue). Group II (Sham treatment): tooth extraction and application of inactive PBM. **Results:** Pain and inflammation decreased similarly in both groups over time. Anxiety decreased in both groups without significant differences (*p* = 0.776; *p* = 0.246). There was no evidence that the treatment or location of the extraction had an influence on healing. Suturing the socket increased the likelihood of good healing (*p* = 0.048), while long procedures reduced it (*p* = 0.040). **Conclusions:** PBM is a non-invasive and safe therapy. This study did not show significant differences with respect to the sham treatment. More research is needed with a standardized methodology to better assess its efficacy.

## 1. Introduction

Surgical procedures in the oral cavity can produce pain and inflammation as side effects, and their intensity will depend on the complexity of the treatment, sensitivity of the patient, and the health professional’s skills, among other factors [1]. A large percentage of patients mention pain during the post-operative period [2]. In studies that have recorded pain after third molar extractions, no relationships have been observed between surgery and the magnitude of post-operative pain [3]. Some authors have observed pain in a third of the patients subjected to oral surgery—more specifically, during the second and third days after the surgery [4,5,6]. Pain, inflammation, and other complications such as a delayed or poor healing have also been described in many works in which minor surgeries were performed in the oral cavity, such as tooth extractions, implants, or periodontal treatments [1,2,6,7]. In order to minimize these effects, pharmacological treatments and hygienic and dietary measures, as well as other care, are prescribed [1]. In addition to the above, another technique has been used, such as Low-Power Diode Laser (LPL), also known as Low-Level Laser Therapy (LLLT) or photobiomodulation (PBM), as it provides benefits after surgical interventions, increasing the healing speed. In addition, it provides an analgesic and anti-inflammatory effect [5,6,7]. Different studies have suggested that the anti-inflammatory effect produced is due to its inhibition of the cyclooxygenase cascade, leading to a reduction in the formation of prostaglandins [7,8]. Photobiomodulation therapy stimulates the formation of fibroblasts and new blood vessels, thus favoring tissue repair and regeneration [8,9,10]. Very few and slight adverse effects have been described, such as a reddening of the treated area or irritation, so that its use has aroused much interest in odontology [7,8,9,10,11,12,13,14,15,16]. However, there is a notable lack of research that has assessed its efficacy in simple extractions, which brings to light the need to address this topic. Even though the surgical trauma after simple extractions is significantly lower, the biological principles of the laser, and its ability to modulate inflammation and promote tissue regeneration, makes us think that it could be equally beneficial in these procedures [16]. Assessing the efficacy of the laser in simple extractions could reduce the need for post-surgery medications and accelerate the recovery of the patient, improving their quality of life [14,15,16]. Additionally, the stress generated in the patient by a dental extraction could lead to inadequate understanding of the post-surgical recommendations, while PBM is an alternative care after surgery that does not require the patient’s attention [17]. The aim of the study was to assess the efficacy of PBM after tooth extractions with respect to pain, inflammation, and healing.

## 2. Materials and Methods

This study was designed as a single-blinded, comparative, randomized, controlled, single-center clinical trial (Table 1) that follows the requirements of the CONSORT statement and the guidelines of the Declaration of Helsinki. The study received a favorable report from the Bioethics Committee of the University of Murcia (ID: 2934/2020; approval date: 16 June 2020) and was conducted in a private dental clinic in the Region of Murcia, Spain. The study was registered at ClinicalTrials.gov, with the following identification number: NCT06943391.

### 2.1. Study Groups

The sample was composed of 124 individuals who had a tooth extraction. Two groups were created with a simple randomization with the free online [18]. Group I (Experimental): Patients subjected to a tooth extraction and immediately given an intraoral PBM session with an EPIC X Biolase diode laser (BIOLASE, Inc., Foothill Ranch, CA, USA) with a voltage of 100–240 V, 1.5 A, a power of 0.5 W, and an application of an energy of 15 J per cm² for 10–30 s at 1 mm of the tissue with a sterile surgical tip based on the optimum biostimulation parameters by Cronshaw et al. [19] and according to the parameters recommended by the manufacturer. It was applied at three points in the area: buccal, palatal/lingual, and occlusal to stimulate the tissue. Group II (Sham): Patients undergoing tooth extraction and the application of inactive/simulated PBM immediately after using the same procedure.

No a priori sample size calculation was performed. The observed effect size for the main variable (pain level) was Cohen’s d = 0.21, suggesting a small effect.

### 2.2. Procedure

#### 2.2.1. a—First Session

All the patients in the study were included after meeting the eligibility criteria and signing the informed consent to participate and to allow the use of the data obtained for research aims. The following data were collected: duration of surgery (minutes), location of the extraction, if sutured, and healing. The following tests and questionnaires were applied:

- Complete clinical and oral evaluation.
- Modified Dental Anxiety Scale by (MDAS) Corah [20,21], with which four ranges were established: <slight or no anxiety, 9–12 moderate anxiety, 13–14 high anxiety, >14 severe.
- Oral Health Impact Profile Questionnaire (OHIP-14sp) [22].

**Figure 1:**
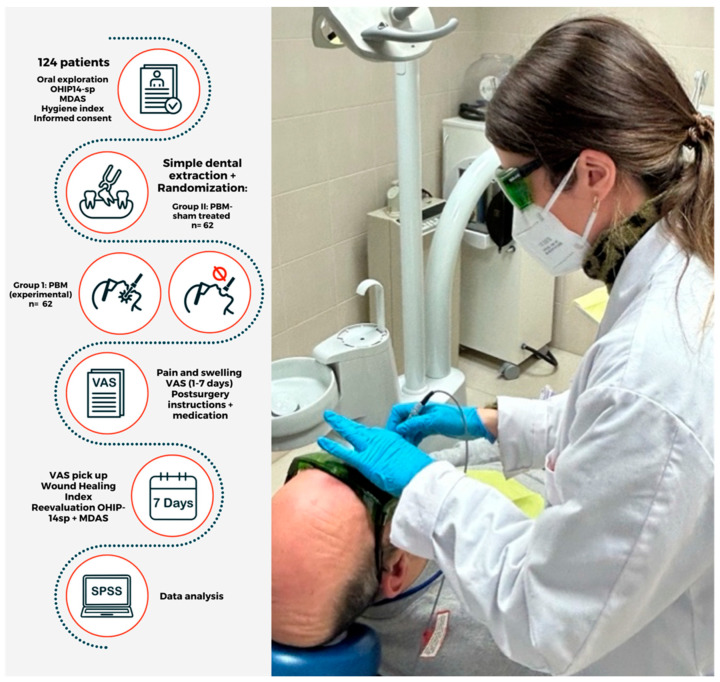
Scheme of the study design.

The tooth was extracted under local anesthesia. The location of the extraction and duration of the procedure were assessed using an EXTECH Stopwatch digital stopwatch (FLIR Commercial Systems, Inc., Wilsonville, OR, USA); after which, LLLT was applied. Post-operative instructions were given, and a questionnaire with a Visual Analog Scale (VAS) was given to assess pain and inflammation in the 7-day post-operative period (Figure 1).

#### 2.2.2. b—Second Visit After 7 Days

- Collection of pain and inflammation questionnaire.
- Evaluation of the healing index according to Hamzani and Chaushu (2018) [23].
- Reevaluation of the MDAS and the OHIP-14sp questionnaire.

#### 2.2.3. Variables (Outcomes)

- Main:

Pain and inflammation, evaluated through the VAS found in the questionnaire during the 7-day post-operational period, with scores ranging from 0 (no pain/no inflammation) to 10 (intense pain/intense inflammation). To evaluate healing, the healing index (HI) by Hamzani and Chaushu (2018) [23] was utilized.

### 2.3. Statistical Analysis

The SPSS (version 29.0) statistical software was used. For pain and inflammation, a repeated-measures ANOVA was performed, with the time measured as the intra-subject factor and the patient group as the inter-subject one. The main effects and interactions were contrasted, and for multiple comparisons, the Bonferroni test was applied. In the case of a binary outcome, a binary logistic regression with odds ratio estimation was used. To assess the homogeneity of the group, with respect to the preoperative variables, and to detect possible confounding variables, chi^2^ tests and *t*-tests for independent samples were performed. To assess the relationship between non-ordinal continuous variables, the Spearman correlation test was used, especially in the analyses in which the ordinal regression could not be adequately estimated. The level of significance used in the analyses was 5% (α = 0.05).

## 3. Results

### 3.1. Descriptive Results and Homogeneity of the Groups

The mean age was 52.5 years, with a standard deviation of 17 years and a range of 18–86 years old. There was a prevalence of endocrine diseases and high blood pressure (Table 2). In the preoperative evaluation, the following were obtained: 50% slight–no anxiety, 20% moderate, 10% high, and 20% severe. With respect to quality of life, the total mean was 13.7. As for location, most of the teeth extracted were posterior (60%). There were no complications associated with the surgical procedure in any of the two groups.

Anxiety was similar in both groups, and the differences between the groups were not statistically significant (*p* = 0.503) (Table 3).

No statistically significant differences were found in the mean level of quality of life in both groups (*p* = 0.854) (Table 4).

### 3.2. Analysis of Pain

Higher levels of pain were observed in the experimental group. Over time, an approximately linear decrease was observed, with a slope that was similar in both groups, with large intra-group variability. To verify this observation, a single-factor repeated-measures ANOVA was performed. The pain significantly decreased over time and, in a similar manner, in both groups on every evaluation day. In the sham group, significant differences were found between days 3 and 4 and days 5 and 6, while, in the experimental group, these were observed between days 2 and 3, days 3 and 4, days 4 and 5, and days 6 and 7.

We were not able to determine if the pain or changes were different in the different locations, not due to use or sutures, duration of the intervention, nor preoperative quality of life.

### 3.3. Analysis of Inflammation

**Figure 2:**
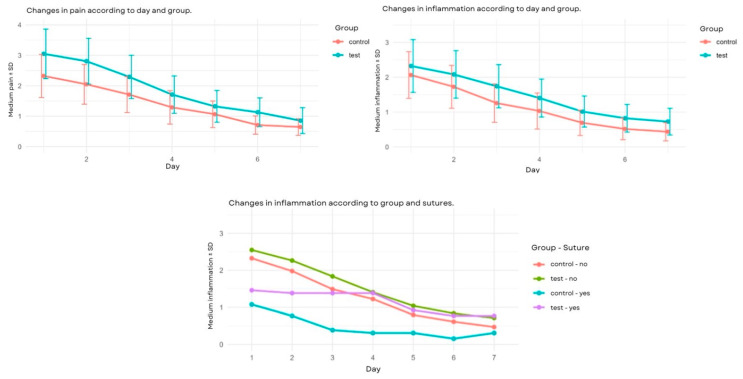
Top left: changes in pain according to day and group. Top right: changes in inflammation according to day and group. Below: changes in inflammation according to group and sutures.

Similar results to pain were found for inflammation: a higher inflammation in the experimental group, a linear decrease over time, and a similar intensity between groups with a great variability in values. No significant evidence was observed that the mean level of inflammation was different between both groups and was extensible to any day. In the sham group, significant differences were only found between days 2 and 3, and in the experimental group, differences were only found between days 3 and 4. No significant differences were observed between the groups (Figure 2). The time x suture interaction was significant (*p* = 0.045) (Table 5), which suggests that the change in inflammation over time varied according to the presence or not of sutures. We cannot attest that there were differences in inflammation due to the duration of the operation or the preoperative quality of life.

### 3.4. Analysis of Post-Operative Complications

To verify the existing relationship between the probability of suffering from complications and belonging to a group, a multiple regression analysis was performed. With a marginal significance (*p* = 0.068), the application of the laser caused bleeding in the experimental group with a probability that was 4.5 times higher than the sham group. The presence of dry socket was not observed in any of the groups.

### 3.5. Analysis of the Healing Index

**Figure 3:**
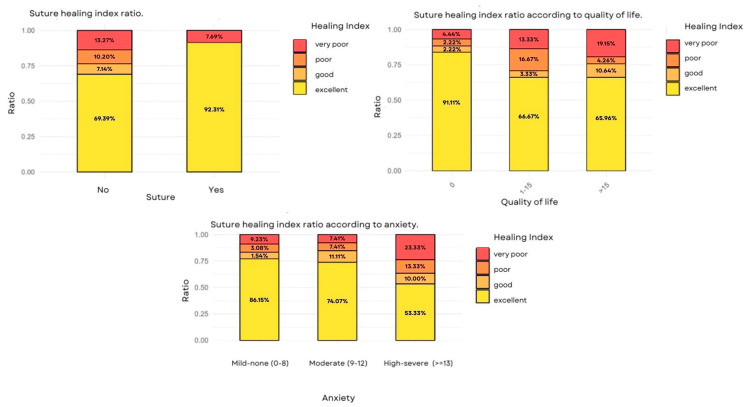
Top left suture healing index ratio. Top right: suture healing index ratio according to quality of life. Below: suture healing index ratio according to anxiety.

The relationship between the probability of better healing and the treatment was analyzed. No statistical evidence was observed that the treatment applied had an effect on healing. There was also no evidence of a relationship with the area of extraction. We found statistical evidence (*p* = 0.048) to affirm that suturing increased the probability of good healing up to 4.5 times higher (OR—4.5) as compared to no sutures (Table 6). It was also observed that, as the surgery time increased, the probability of good healing decreased. There was enough statistical evidence (*p*—0.040) to state that operations longer than 15 min were associated with a lower probability of better healing, reducing it by 65% (OR = 0.348) as compared to operations lasting 10 or less than 10 min (Figure 3).

The ratio of excellent healing was reduced as anxiety increased and the quality of life worsened. There was enough statistical evidence to conclude that severe levels of anxiety and a poor quality of life were associated with a lower probability of good healing, reducing it by 81% (OR = 0.191) in both cases.

## 4. Discussion

Photobiomodulation is a non-invasive therapy without side effects for patients, which could reduce the intake of fast-acting medicines [1,5,6]. The results from our study indicate that PBM with a Low-Power Diode Laser did not show significant clinical benefits in simple tooth extractions.

In more complex procedures, such as third molar extractions, PBM has been shown to be more effective [6,13,24,25,26,27,28,29]. These interventions tend to imply a greater surgical manipulation of the bone and soft tissue, which generates a more intense inflammatory process and more severe post-operative pain. In addition, healing is slower due to the greater tissue damage, so that the biostimulation effect of the PBM on cellular regeneration and the modulation of inflammation becomes more relevant [14].

It was found that patients who were given sutures were 4.6 times more likely to experience good healing as compared to those without sutures (OR = 4.634, *p* = 0.048). This suggests that sutures help protect blood clotting, promoting better tissue regeneration [26]. Procedures lasting more than 15 min reduced the probability of good healing by 65% (OR = 0.348, *p* = 0.040). Longer surgeries could generate more surgical trauma and more inflammation, affecting recovery.

The quality of life, and fear and anxiety of dental treatment, are factors that must be considered in the post-operational period of a patient. Patients with severe preoperational anxiety and poor quality of life had an 81% reduction in the probability of good healing (OR = 0.191, *p* < 0.04). Thus, stress can affect the inflammatory response and the immunological system, which could compromise the healing process. In the study by Niemczyk et al. [17], those patients who underwent complex extractions had higher levels of stress, independent of the pain levels. According to Souza et al. [27], most people experience a significant deterioration in oral health-related quality of life (OHRQoL) in the first few days after a tooth extraction. In the present study, we evaluated OHRQoL through the HOIP-14 questionnaire at the start and during the 7-day post-operative period, without finding differences between the treatment and sham groups. This lack of differences could be due to the limited follow-up period, the fast recovery that is characteristic of simple extractions, and the individual variability in the perception of the patient.

In the study by Tenis et al. [28], the authors obtained good results with the application of PBM after wisdom tooth extractions. In the study by de Metin et al. [29], in turn, the authors used PBM after performing other oral surgical interventions, obtaining improvements in quality of life, pain, and post-operational follow-up of the patients.

The location where the laser was applied had an influence on the results. Ahrari et al. [30] found that the intraoral application favored healing but did not significantly reduce pain, a finding that is in agreement with the results by Pol et al. [24] and with those obtained in the present study. On the other hand, Momeni et al. [31] suggested that the extraoral or combined application is more effective, showing better clinical results.

The present study had some limitations that must be considered when interpreting the results. The post-operative follow-up was limited to seven days, which could have been insufficient to detect later effects of PBM in healing and quality of life. Although the sample was adequate, non-controlled effects, such as oral hygiene and adhesion to post-operational recommendations, could have influenced the clinical outcomes. Future studies could lengthen the follow-up period and optimize the parameters through standardized protocols to improve the understanding of the impact of PBM in the post-surgery recovery process.

PBM did not have a significant effect on healing after simple extractions, perhaps because the recovery process in these cases is fast and efficient by itself. However, sutures were identified as a key factor in improved healing, while longer interventions were associated with poor healing. A comprehensive approach that includes anxiety monitoring, an adequate hygiene, and a reduction in the duration of the surgery could favor better results in the patient’s recovery.

## 5. Conclusions

These results show that PBM did not show significant differences with respect to the sham treatment (PBM-Sham) in terms of pain, inflammation, and healing. More studies with a standardized methodology are needed to evaluate the efficacy of the therapy after simple extractions.

## Figures and Tables

**Table 1:** Inclusion and exclusion criteria.

Inclusion Criteria	Exclusion Criteria
1. Adult patients (>18 years)	1. ASA preoperative evaluation: bad conditions for surgery
2. Need of tooth extractions (for any reason)	2. Inmunocompromised patients or taking inmunosuppressants
3. Signed an informed consent form	3. Patients with decompensated systemic diseases
	4. Patients undergoing chemotherapy treatment
	5. Pregnant women
	6. Patients with several mental disorders
	7. Patients who had received head and neck radioteraphy

**Table 2:** Description of the most important demographic and clinical characteristics.

	Group		*p*-Value
	Sham (*n =* 62)	Experimental (*n* = 62)	
Sex			0.590
Male	31 (50%)	28 (45.2%)	
Female	31 (50%)	34 (54.8%)	
Age (years)	51.4	53.5	0.490
Duration of Surgery (minutes)	13	14.1	0.245
Location			0.814
Anterior area	8 (12.9%)	10 (16.1%)	
Middle area	13 (21%)	13 (21%)	
Posterior area	37 (59.7%)	36 (58.1%)	
Posterior middle area	1 (1.6%)	2 (3.2%)	
Anterior middle area	3 (4.8%)	1 (1.6%)	
OHIP TOTAL (Day 1)	14.6	12.7	0.854
MDAS TOTAL (Day 1)	7.9	7.7	0.503
SUTURES			1
Yes	13 (21%)	13 (21%)	
No	49 (79%)	49 (79%)	

OHIP: Impact of Oral Health of Quality. MDAS: Modified Dental Anxiety Scale.

**Table 3:** MDAS scale on day 1 (basal) and day 7 (post-treatment).

		GROUP			*p*-Value
		TOTAL	Sham	Experimental	
MDAS BASAL (T1)	Total	124 (100%)	62 (100%)	62 (100%)	0.503
	Slight-none (0–8)	66 (53.2%)	33 (53.2%)	33 (53.2%)	
	Moderate (9–12)	28 (22.6%)	14 (22.6%)	14 (22.6%)	
	High (13–14)	7 (5.6%)	3 (4.8%)	4 (6.5%)	
	Severe (>15)	23 (18.5%)	12 (10.4%)	11 (17.7%)	
MDAS POST (T2)	Total	124 (100%	62 (100%)	62 (100%)	
	Slight-none (0–8)	91 (73.4%)	47 (75.8%)	44 (71%)	
	Moderate (9–12)	19 (15.3%)	9 (14.5%)	10 (16.1%)	
	High (13–14)	2 (1.6%)	1 (1.6%)	1 (1.6%)	
	Severe (>15)	12 (9.7%)	5 (8.1%)	7 (11.3%)	

MDAS: Modified Dental Anxiety Scale. T1: time 1. Post: post-treatment.

**Table 4:** OHIP14-SP scale on day 1 (basal) and day 7 (post-treatment) and the differences between them.

		GROUP			
		TOTAL	SHAM	Laser	*p*-Value
OHIP14-SP (T1)	*N*	124	62	62	0.854
	Mean	13.7	14.6	12.7	
	Standard deviation	15.9	18.5	12.8	
OHIP14-SP (T2)	*N*	124	62	62	
	Mean	8.9	7.5	10.2	
	Standard deviation	14.7	14.8	14.5	

OHIP: Impact of Oral Health of Quality. T1: time 1. T2: post-treatment.

**Table 5:** Changes in inflammation according to group and sutures: results from the F-test of the repeated-measures ANOVA model.

	F	*p*-Value
TIME	17.733	<0.001 ***
GROUP	1.273	0.261
SUTURE	1.995	0.160
GROUP × SUTURE	0.272	0.603
GROUP × TIME	0.332	0.715
TIME × SUTURE	3.158	0.045 *
GROUP × SUTURE × TIME	0.293	0.743

* *p* < 0.05; *** *p* < 0.001.

**Table 6:** Probability of having better healing depending on the suture: results of the multiple logistic regression model: odds ratio (OR) and 95% confidence interval.

	OR	95% CI	*p*-Value
GROUP			
Control	1		
Test	0.539	0.22–1.26	0.157
SUTURE			
No	1		
Yes	4.634	1.24–30.33	0.048

## Data Availability

The raw data supporting the conclusions of this article will be made available by the authors on request.
